# Person-centred, occupation-based intervention program supported with problem-solving therapy for type 2 diabetes: a randomized controlled trial

**DOI:** 10.1186/s12955-020-01521-x

**Published:** 2020-08-03

**Authors:** Zeynep Bahadır Ağce, Gamze Ekici

**Affiliations:** 1 Department of Occupational Therapy, Uskudar University, İstanbul, Turkey; 2 Department of Occupational Therapy, Hacettepe University, Ankara, Turkey

**Keywords:** Diabetes mellitus, Person-centred, Occupation-based, Problem-solving, Psychosocial self-efficacy, Coping, Well-being, Quality of life, Occupational therapy

## Abstract

**Background:**

Individuals with diabetes mellitus have difficulty solving problems in meaningful occupations and have similar difficulties with self-care regimens. We examined the effects of an occupation-based intervention supported with problem-solving therapy in individuals with type 2 diabetes mellitus on participation in and satisfaction with meaningful occupations, diabetes-related psychosocial self-efficacy, preferred coping strategies and individual well-being.

**Methods:**

This study was planned as a single-blind, randomised controlled study with a 3-month follow-up involving 67 adults with type 2 diabetes. The Canadian Occupational Performance Measure, Diabetes Empowerment Scale, Brief COPE and five-item World Health Organisation Well-Being Index were used. This programme included evaluations, diabetes education, and problem-solving therapy. The intervention was conducted for 6 weeks, and each weekly session lasted approximately 60 min. Differences between groups were analysed using the Mann-Whitney U test, and the Friedman test was used to calculate group-time interaction differences (i.e., baseline, after 6 weeks and after 3 months).

**Results:**

All participants identified the most significant occupational performance problems in self-care as personal care. Significant improvement was reported in the intervention group compared to the control group regarding participation in meaningful occupation, satisfaction with performance, psychosocial self-efficacy, and well-being results (*p* < 0.001) after the programme and 3 months of follow-up. Participant use of effective coping strategies, active coping and acceptance strategies, and self-efficacy, as revealed by the results, suggested improvement in favour of the intervention group (*p* < 0.05).

**Conclusions:**

Occupation-based problem-solving therapy encourages participation in meaningful occupations and improves psychosocial self-efficacy, effective coping styles, and well-being in patients with type 2 diabetes mellitus. Problem-solving therapies that incorporate individuals’ priorities via meaningful occupation can be used to lead to a meaningful and quality life for individuals with type 2 diabetes mellitus.

**Trial registration:**

ClinicalTrials.gov Identifier: NCT03783598. Retrospectively Registered. First Posted-December 21, 2018, Last Update Posted-February 18, 2020.

## Background

Occupations and occupational performance are a focus of occupational therapy practice because they affect health conditions [1, 2]. Occupation covers all the activities that people do to occupy themselves and bring meaning and purpose to life, such as looking after themselves, enjoying life, and contributing to the social and economic fabric of their communities [3]. Participation in the everyday occupations of life is vital for humans, and an individual’s ability to carry out everyday occupations (occupational performance) has positive effects on health and well-being [2, 4]. Each person has different characteristics that combine to produce specific occupations by particular clients in singular environments; therefore, the person-centred approach accepts the client’s uniqueness [5]. Person-centred approaches have a holistic perspective incorporating contextual factors, such as daily self-management experiences and cultural habits of the individual, and facilitate the performance of everyday tasks and adaptation to settings in which the person works, lives and socialises [3].

Diabetes mellitus is a chronic disease that negatively affects every aspect of a person’s life, and individuals may complain about some difficulties when participating in occupations such as diabetes self-care, work or social engagements [6]. Diabetes mellitus especially affects the participation in meaningful occupations by individuals [7]. The participation of individuals in meaningful occupations is important in fostering meaning in life [8]. However, there is substantial evidence showing better motivation and participation in the recovery process when individuals are engaged in meaningful occupations [9]. Thus, it is important to investigate the occupational performance problems of individuals according to their meaningful and valuable priorities [10, 11]. Individuals can sustain through their pursuit of meaningful occupations and maintain a personally meaningful lifestyle [12].

Diabetes mellitus, which is one of the most complex chronic diseases, has a continuously changing dynamic structure and causes individuals to struggle with many obstacles at the same time [13–15]. Healthy living with diabetes mellitus is a challenging process influenced by many personal and environmental factors [16]. Individuals are advised to continue with their ordinary life while carrying out their self-care for diabetes to maintain a healthy life [17, 18]. Sometimes, participation in multiple occupations may lead to poorer health for individuals due to complexity [2]. Individuals sometimes encounter conflicts between participating in activities that are meaningful to them and their diabetes self-management behaviour, and these situations may cause stress [19]. A person needs to cope with stress, spiritual status, responsibility level, knowledge of diabetes, demands of complex situations, and the relationship between the individual’s cultural, physical and social environments [14, 16, 20–22]. Problem-solving therapy (PST) can be an effective way of developing the coping skills of individuals with type 2 diabetes [23].

PST is a cognitive behavioural intervention developed by D’Zurilla and Goldfried to alleviate individuals’ mental and physical problems and improve their ability to cope with stressful life experiences [24]. PST is considered a guiding method to clarify the goals of individuals and develop alternative solutions [25]. PST helps support the individual with a disease or disability to overcome the barriers of participation that develop as a result of the problems experienced at home and in the community [25–27]. It is recommended that problem-solving interventions be adapted to include individual needs for the person with diabetes [28]. PST has been used in many chronic disorders, but it has not been implemented through meaningful occupation problems by individuals in the context of occupational therapy [29, 30].

This study is based on the lack of adequate research on PST applied through meaningful occupation problems and assessed by the researcher. In the current study, we tested the hypothesis that occupation-based PST could (i) increase ratings of performance and satisfaction with meaningful occupations, (ii) increase ratings of diabetes-related psychosocial self-efficacy, (iii) improve ratings of effective coping strategies, and (iv) enhance ratings of well-being.

## Methods

### Study design and description of the participants

This study was designed as a single-blind, randomised controlled trial. The sample size was determined using 5% (*p* = 0.05) Type 1 error, 80% strength through statistical power analysis, and a two-way hypothesis testing and consisted of 33 subjects per group. Participants for this study were selected from individuals who presented at the internal medicine outpatient department of a state hospital in Turkey. Individuals who were diagnosed with type 2 diabetes mellitus, were aged between 18 and 65 and were literate in Turkish were included in this study, and individuals with a diagnosis of a mental illness or cancer were excluded from this study. A total of 86 individuals were referred to this study.

**Fig. 1:**
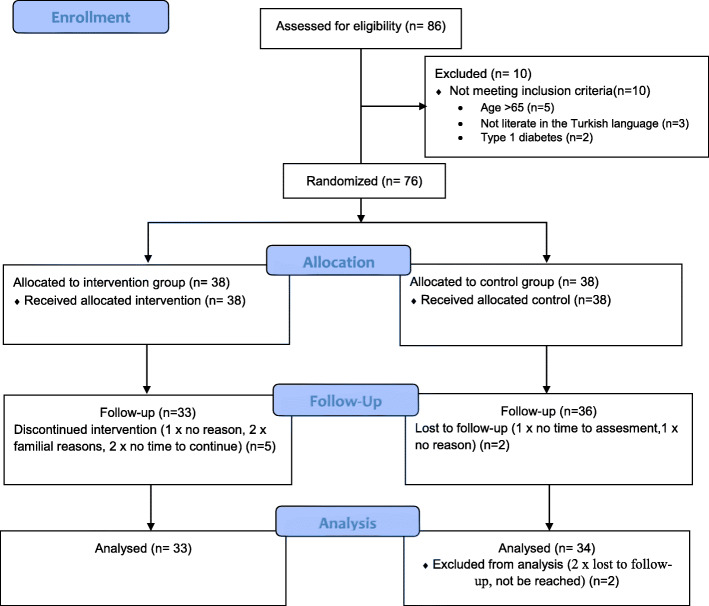
The CONSORT Flow Diagram Chart of Enrollment

Eligibility assessments of individuals were taken before allocation to the study groups. Among the 86 referred individuals, 10 were excluded from this study because they did not meet the inclusion criteria. The remaining 76 individuals were randomly assigned (using the simple random number table) to either the intervention group or control group (*n* = 38 each). For the control and intervention groups, randomisation was applied separately using stratification variables produced by the website Research Randomizer (https://www.randomizer.org/#randomize). A simple random number table was created for each group. Numerical distribution was ensured as 2 sets of unique numbers were formed. The participants were assigned to the groups according to the randomisation number. The baseline evaluation was carried out at the first meeting that was individually planned for the participants in each group after randomisation. Nine subjects discontinued participation for various reasons during the process, and this study was therefore completed with 67 individuals with type 2 diabetes. Figure 1 shows the CONSORT flow diagram of this study.

All subjects gave consent after they were provided with verbal and printed information about this research project. Our study was conducted in accordance with the Helsinki Declaration (revised in 2013) after ethical approval was obtained (approval number: GO15/731). This study was conducted between June 2015 and September 2017.

The scales described in detail below were completed by all participants. Participants in both groups completed the scales face-to-face with the same therapist who provided guidance. The individuals were assessed at baseline, after 6 weeks, and after 3 months.

### Measurements

#### Socio-demographic and clinical features form

Each participant was asked to complete a form to obtain demographic information, such as age, gender, marital status, health-related habits (e.g., smoking and alcohol consumption), body mass index (BMI) and medical history (e.g., monitoring, family history, and comorbidity).

### Primary outcomes

#### The Canadian occupational performance measure (COPM)

The COPM is based on a semi-structured interview method and helps individuals identify and prioritise activities of importance that they have difficulty performing [31]. The COPM can be used as both an intervention and an assessment tool. Specifically, the COPM is a measure of self-perceived occupational performance areas in self-care (personal care, functional mobility and community management), productivity (paid/unpaid work, household management and play/school) and leisure (quiet recreation, active recreation and socialisation). First, the importance of each occupation was rated on a 10-point scale where 1 is “not important” and 10 is “extremely important”. Then, up to five of the most important activities were rated for performance from 1 (“do not perform well”) to 10 (“perform very well”) and for satisfaction from 1 (“not satisfied”) to 10 (“very satisfied”). The obtained performance and satisfaction scores were separately collected and divided by the number of activities to obtain a performance score and a satisfaction score [32]. The test-retest reliability of the COPM is within the acceptable range; intraclass correlation and obtained coefficients for individuals with chronic diseases ranged from scores of 0.86 to 0.89 for performance and 0.76 to 0.88 for satisfaction [33–35]. The COPM has shown validity and reliability in Turkish samples [36]. The COPM is recommended for use in diabetic subjects to identify what is important and their priorities [10].

### Secondary outcomes

#### The diabetes Empowerment scale (DES)

The DES is used in the measurement of diabetes-related psychosocial self-efficacy and consists of 28 items with three subscales as follows: managing the psychosocial aspects of diabetes; assessing dissatisfaction and readiness to change; and setting and achieving diabetes goals [37]. Each question is rated between 1 for “strongly disagree” and 5 for “strongly agree”. Thus, higher scores indicate better psychosocial self-efficacy levels. The DES is a valid and reliable scale in Turkish populations [38].

#### The brief COPE

The Brief COPE measures strategies for coping with stress and includes 14 subscales in which two items are grouped into two coping strategies: effective approach coping (active coping, acceptance, positive reframing, planning, use of emotional or instrumental support) and ineffective avoidant coping (denial, self-distraction, substance use, behavioural disengagement, venting and self-blame) [39]. It is uncertain whether the humour and religion subscales are effective or ineffective coping styles; therefore, they were excluded from both analyses [40]. Each question has a selection range from 1 (“I have not been doing this at all”) to 4 (“I have been doing this a lot”), and the higher subscale scores indicate using those coping strategies more. These tools are also valid and reliable in Turkish populations [41].

#### The World Health Organisation-five well-being index (WHO-5)

The WHO-5 is used for the psychometric evaluation of emotional well-being, depression, and quality of life. This measure consists of five statements, which respondents rate on a scale of 0 (“never”) to 5 (“all the time”) considering the previous 2 weeks [42]. The raw value, ranging from 0 to 25, is multiplied by four to determine the final score, with 0 representing the worst possible well-being and 100 the best. The WHO-5 has Turkish validity and reliability [43, 44], and a cut-off of less than 50% has been identified to screen for depression and reduced well-being [45].

### Intervention programme

The complex nature of diabetes mellitus affects individuals’ participation in life in different ways. This intervention programme is designed to use PST to overcome the problems of participation in meaningful occupations defined by individuals.

This intervention programme included the four steps of PST: (1) problem definition, (2) generation of alternatives, (3) decision-making, and (4) solution implementation and verification [46]. In particular, the intervention programme considered the demands and priorities of the person at the stage of “problem definition” and the self-perceived occupational performance problems that were meaningful to the person.

This programme, which includes assessment tools, education, and PST, was implemented by the same therapist who has a cognitive behavioural therapy certificate and 3 years of PST experience. The intervention programme was 6 weeks in duration with one session a week, and each session lasted approximately 60 min. The intervention was face-to-face and individually held in a clinical setting at a suitable time for the person. The content of the six-week programme is explained in detail below.

- Week 1: The purpose of the first session was for the intervention group participant to complete all measurements together with the therapist. The therapist also explained how to complete the diary on a typical day. The therapist and participants together identified the problematic activities with the COPM. The COPM allowed them to identify the individuals’ goals regarding performance problems in their meaningful activities. The COPM was used as both an assessment and an intervention tool for the intervention group.

In addition, the first week is equivalent to the “problem definition” step of PST for the intervention group. Briefly, this step included setting a measurable, realistic, and attainable goal for the solution in the first week [47].

- Week 2: The purpose of this session was to provide education with information about diabetes mellitus. The education was delivered one-to-one through a PowerPoint presentation. This educational information follows the National Standards for Diabetes Self-Management [48] and is based on a Person-Environment-Occupation Model [49]. We, therefore, focused on therapeutic lifestyle changes that include basic knowledge and skills relating to diabetes as well as personal, environmental, and occupational factors that affect the condition. The emphasis was on the elements of daily life that could support or prevent effective diabetes management and the importance of identifying these elements.
- Week 3: In this session, the “generation of alternatives” and “decision making” step of PST was applied. This step included the generation of alternatives to possible solution strategies and assessments of the advantages or disadvantages of each strategy related to the action [47]. The best solution strategy for the occupational performance problem that was defined in the first week with the help of the COPM was determined. To determine the best strategy for overcoming obstacles, alternative solutions were explored through brainstorming, and an action plan was created once the most appropriate approach had been identified. The individual was encouraged to recognise and use their environmental and personal resources. In addition, the completed diary was used to determine the steps to adopt time, frequency and duration aspects of the action plan into a daily schedule.
- Weeks 4 and 5: These sessions involved the “solution implementation and verification” step of PST. Moreover, it included the implementation of the strategies and verification of the solution [47]. The action plan was reviewed by sharing the individual’s experiences, and possible alternatives and new strategies were defined when necessary. Together, they discussed when the solution plan was carried out, the individual, environmental, supportive, and preventive factors that were involved, the consequences of the plan and whether problem-solving efforts had been successful or needed revision.

Sample questions of sharing the action plan experience were as follows: What were opportunities or obstacles in the action plan experience? How did the individual approach unpredictable developments? Which events were coped with in a good or bad way? How could it be possible to reach the target in a different way?

The action plan was revised to consider an individual’s requirements when needed.

- Week 6: In the last session, all questionnaires were completed again. In addition, the last session included the sharing of experiences and the discussion of future goals.

### Statistical method

Data were analysed using IBM Statistical Package for the Social Sciences (SPSS) version 21.0 software [50]. Missing values were excluded from that analysis. A multiple regression analysis based on the change score on the COPM, Brief COPE, DES and WHO-5 after controlling for gender, occupational status, diabetes duration and treatment regime. To use controlling variables in the regression analysis, dummy variables were produced for ordinal variables. The Shapiro-Wilk test was used to evaluate the distribution of the collected data (normal = *p* > .05, not normal = *p* < .05). The chi-square and Mann-Whitney U tests were used to assess differences in demographic variables between the groups (p < .05). Differences between the groups were analysed with independent t-tests (parametric) or Mann-Whitney U tests (non-parametric). Group-time interaction differences (i.e., baseline, after 6 weeks and after 3 months) were calculated using the Friedman test. The level of significance was set at 0.05. Quantitative variables are expressed as the mean ± standard deviation (X ± SD), and qualitative data are described with percent (%) values.

Thematic analysis was used to evaluate the qualitative data of activities (COPM), which were presented as percentages (%). The COPM data were categorised according to performance areas with the MAXQDA code system, and percentage data were obtained. Coding was conducted with MAXQDA 11.0 [51] through which the qualitative data were coded as self-care, productivity or leisure to develop a picture of occupational performance across all areas of life.

## Results

Demographic variable analysis showed that the mean age of the participants diagnosed with type 2 diabetes mellitus was 54.64 (±8.93) years in the intervention group (IG) and 55.76 (±8.16) years in the control group (CT). The participants stated that they exercised regularly, with a rate of 26.5% in the CT and 12.1% in the IG. The mean weekly exercise of the participants was 1.75 (± 2.6) days in the IG and 2.47 (± 2.32) days in the CT. There were no significant differences between the demographic variables, as seen in Table 1.

**Table 1:** Demographic information of the intervention and control groups

	Intervention group (IG)		Control group (CG)		
	Mean (min max) ± SD		Mean (min max) ± SD		p* values
**Age**	54.64 (37–65) ± 8.937		55.76 (35–65) ± 8.16		0.591
**Body mass index (BMI) kg/m**^**2**^	31.22 (19.4–47.5) ± 6.62		29.1 (19.03–45.1) ± 5.24		0.151
**Monitoring in weekly (times)**	3.55 (0–14) ± 5.33		4.87 (0–14) ± 5.1		0.153
**Exercises in weekly (day)**	1.75 (0–7) ± 2.6		2.47 (0–7) ± 2.32		0.097
	**n**	**%**	**n**	**%**	***p*values**
Gender					
**-female**	19	57.6	23	67.6	0.394
**-male**	14	42.4	11	32.4	
Occupation					
**- yes**	12	36.4	4	11.7	**0,018**
**-no**	21	63.6	30	88.3	
Marital status					
**-married**	27	81.8	28	82.4	0.954
**-single**	6	18.2	6	17.6	
Alcohol consumption					
**-yes**	4	12.1	3	8.8	0.659
**-no**	29	87.9	31	91.2	
Smoke					
**-yes**	7	21.2	7	20.6	0.950
**-no**	26	78.8	27	79.4	
Monitoring					
**-yes**	14	42.4	18	52.9	0.389
**-no**	9	57.6	16	47.1	
Family history					
**-yes**	24	72.7	25	73.5	0.941
**-no**	9	27.3	9	26.5	
Regular exercise					
**-yes**	4	12.1	9	26.5	0.141
**-no**	29	87.9	25	73.5	
Comorbidity					
**-yes**	16	48.5	12	35.3	0.274
**-no**	17	51.5	22	64.7	

p* Man Whitney U Test**,** p Chi-Square Test

Univariate and multivariate regression analysis results in both the CT and IG were significant for ineffective and effective coping change scores (*p* < 0.05). The direction of the relationship was positive for COPM-performance differences for both effective and ineffective coping scores. Regression coefficients showed that the effective coping score was more effective than the ineffective coping score in both the CT and IG. Effect sizes for both ineffective and effective coping scores were higher in the CT than in the IG. The results are given in Table 2.

**Table 2:** Univariate and multivariate regression analysis for research parameter changes

	Univariate			Multivariate		
	B	t	p	B	t	p.
Intervention						
(Constant)				4.092	0.853	0.405
COPM-Performance Change	−1.312	− 1.539	0.134	− 0.615	− 0.710	0.486
COPM-Satisfaction Change	0.215	0.302	0.765	−0.876	− 1.105	0.283
DES Change	1.054	0.254	0.801	−0.084	−0.028	0.978
Ineffective COPING Change	1.518	6.261	**0.000**	0.919	2.790	**0.012**
Effective COPING Change	1.196	4.499	**0.000**	1.149	3.332	**0.004**
WHO-5 Change	−0.124	−1.574	0.126	−0.026	− 0.431	0.671
Gender	−0.538	−0.154	0.879	−0.016	− 0.005	0.996
Occupation	0.131	0.036	0.971	2.668	0.815	0.425
Treatment history	−1.860	−0.541	0.593	−3.691	−1.411	0.174
Diabetes duration (0–10 years)	−0.733	−0.211	0.834	−1.108	−0.352	0.729
Diabetes duration 2 (11–20 years)	1.511	0.358	0.723	−0.393	−0.092	0.928
Diabetes duration 3 (21–30 years)	18.906	1.992	0.055	−1.789	−0.203	0.841
**R**^**2**^**:**0.619; **F:**5.192; p < 0.05						
Control						
(Constant)				0.675	0.442	0.663
COPEM Performance Change	−0.756	−0.706	0.485	−0.264	− 0.533	0.600
COPEM Satisfaction Change	0.033	0.033	0.974	−0.222	−0.592	0.560
DES Change	0.290	0.075	0.941	−2.368	−1.539	0.139
Ineffective COPING Change	1.518	6.366	**0.000**	1.104	7.506	**0.000**
Effective COPING Change	1.395	8.249	**0.000**	1.058	9.618	**0.000**
WHO-5 Change	−0.027	− 0.365	0.717	−0.017	− 0.556	0.584
Gender	−0.198	−0.059	0.953	−1.900	−1.293	0.210
Occupation	−3.217	−0.670	0.508	0.631	0.281	0.781
Treatment history	−1.280	−0.394	0.697	−0.370	−0.246	0.808
Diabetes duration (0–10 years)	−2.643	−0.844	0.405	−0.978	− 0.574	0.572
Diabetes duration 2 (11–20 years)	2.500	0.685	0.498	−0.718	−0.334	0.742
Diabetes duration 3 (21–30 years)	2.485	0.270	0.789	3.786	0.924	0.366
**R**^**2**^**:**0.899; **F:**25.436; p < 0.05						

The COPM-performance qualitative data coded with MAXQDA indicated that both groups had the most difficulty in self-care activities, followed by leisure time and productivity activities, as illustrated in Table 3. The COPM-performance and COPM-satisfaction baseline scores in the CT (3.51 ± 2.11 and 5.25 ± 2.61, respectively) were better than those in the IG (2.51 ± 1.19 and 2.93 ± 1.42). Initially, when the COPM data were compared using a Mann-Whitney U test, the findings showed that differences regarding performance in and satisfaction with occupation between the groups was in favour of the CT. However, at the end of the intervention programme and at the three-month follow-up, the COPM-performance and the COPM-satisfaction scores had significantly increased in the IG. Friedman tests showed an improvement in COPM performance and satisfaction scores after the intervention and after 3 months, that is, the improvement occurred over time in the IG (COPM performance: χ^2^ = 45.690, *p*<0.001; COPM satisfaction: χ^2^ = 41.081, *p*<0.001) but not in the CT (COPM performance: χ^2^ = 0.485, p>0.05; COPM satisfaction: χ^2^ = 1.040, *p*>005). Details are shown in Table 4.

**Table 3:** Occupational performance problems defined by individuals according to performance areas

The Canadian Occupational Performance Measure	Intervention group %	Control group %
SELF CARE TOTAL	**71.82**	**73.01**
-Personal care	45.5	60.3
-Functional mobility	5.44	3.17
-Community management	20.88	9.51
PRODUCTIVITY TOTAL	**1.81**	**1.59**
-Paid/unpaid work	0.9	1.59
-Household management	0.9	0
-Play/school	0	0
LEISURE TOTAL	**26.37**	**25.4**
-Quiet recreation	0	0
-Active recreation-being active	21.83	20.64
-Socialization	4.54	4.76

MAXQDA 11.0, Coded as occupational performance area and calculated as percentage

**Table 4:** Comparison of the intervention and control groups inside and between themselves

		Intervention group	Control group	Comparison of groups	
		Mean ± SD	Mean ± SD	z	p*
CANADIAN OCCUPATIONAL PERFORMANCE MEASURE (COPM)					
COPM-Performance	BI	2.51 ± 1.19	3.51 ± 2.11	−2.1	**0.03**
	AI	6.03 ± 2.13	3.58 ± 2.45	−4.11	**0.00**
	AI-3MNT	6.44 ± 2.21	3.48 ± 2.01	−4.74	**0.00**
	χ^2^; p**	45.690; **0.000**	0.485; 0.784		
COPM-Satisfaction	BI	2.93 ± 1.42	5.25 ± 2.61	−3.68	**0.00**
	AI	7.19 ± 2.23	4.83 ± 2.70	−3.39	**0.00**
	AI-3MNT	7.44 ± 2.37	4.4 ± 2.11	−4.64	**0.00**
	χ^2^; p**	41.081; **0.000**	1.040; 0.595		
DIABETES EMPOWERMENT SCALE (DES)					
Psychosocial aspects	BI	3.19 ± 0.63	3.79 ± 0.64	−3.43	**0.00**
	AI	3.95 ± 0.73	3.67 ± 0.41	−2.20	**0.02**
	AI-3MNT	4.36 ± 0.58	3.7 ± 0.43	−4.66	**0.00**
	χ^2^; p**	52.452; **0.000**	0.578; 0.749		
Dissatisfaction and readiness to change	BI	3.37 ± 0.45	3.81 ± 045	−3.53	**0.00**
	AI	3.93 ± 0.5	3.76 ± 0.23	− 0.96	0.33
	AI-3MNT	4.25 ± 0.32	3.66 ± 0.4	−5.49	**0.00**
	χ^2^; p**	41.785; **0.000**	3.038; 0.219		
Setting and achieving diabetes goals	BI	3.32 ± 0.74	3.87 ± 0.42	−3.47	**0.00**
	AI	4.28 ± 0.65	3.85 ± 0.36	− 3.15	**0.00**
	AI-3MNT	4.49 ± 0.5	3.74 ± 0.4	−4.77	**0.00**
	χ^2^; p**	46.934; **0.000**	2.032; 0.362		
DES-total	BI	3.31 ± 0.54	3.82 ± 0.44	−3.80	**0.00**
	AI	4.07 ± 0.55	3.76 ± 0.26	−2.35	**0.01**
	AI-3MNT	4.46 ± 0.74	3.7 ± 0.35	−5.33	**0.00**
	χ^2^; p**	53.786; **0.000**	2.305; 0.316		
BRIEF COPE					
Ineffective coping	BI	26.27 ± 5.09	27.56 ± 4.64	−1.038	0.299
	AI	26.55 ± 5.01	27.09 ± 5.4	−0.592	0.554
	AI-3MNT	25.09 ± 4.31	27.03 ± 4.39	−2.007	**0.045**
	χ^2^; p**	0.638; 0.72	0.331; 0.84		
Self-distraction	BI	6.45 ± 1.76	6.26 ± 1.44	− 0.95	0.33
	AI	5.94 ± 1.56	6.41 ± 1.45	−1.43	0.15
	AI-3MNT	5.58 ± 1.56	6.71 ± 1.21	−3.05	**0.00**
	χ^2^; p**	9.484; **0.009**	2.064; 0.356		
Denial	BI	2.79 ± 1.61	3.74 ± 1.67	−2.79	**0.05**
	AI	3.33 ± 1.89	3.88 ± 1.71	− 1.37	0.16
	AI-3MNT	2.85 ± 1.52	3.44 ± 1.39	1.94	0.051
	χ^2^; p**	0.974; 0.615	2.849; 0.241		
Substance use	BI	2.94 ± 2.03	3.29 ± 1.94	−1.24	0.21
	AI	2.97 ± 2.06	2.82 ± 1.80	−0.08	0.93
	AI-3MNT	2.67 ± 1.84	3.09 ± 2.06	−1.32	0.18
	χ^2^; p**	2.077; 0.354	1.565; 0.45		
Behavioral disengagement	BI	2.82 ± 10.7	3.68 ± 1.62	−2.29	**0.02**
	AI	2.48 ± 1.06	3.35 ± 1.32	−2.29	**0.03**
	AI-3MNT	3.27 ± 1.73	3.56 ± 1.63	−0.89	0.36
	χ^2^; p**	8.605; **0.014**	1.723; 0.422		
Venting	BI	5.42 ± 1.92	5.53 ± 1.54	−0.14	0.88
	AI	6 ± 1.69	5.94 ± 1.53	−0.18	0.85
	AI-3MNT	5.39 ± 1.51	5.79 ± 1.40	−1.09	0.27
	χ^2^; p**	1.089; 0.580	3.519; 0.172		
Self-blame	BI	5.85 ± 1.87	5.06 ± 1.27	−1.81	0.06
	AI	5.82 ± 1.81	4.68 ± 1.60	−2.69	**0.00**
	AI-3MNT	5.33 ± 1.83	4.44 ± 1.44	−2.12	**0.03**
	χ^2^; p**	2.931; 0.231	2.902; 0.234		
Effective coping	BI	29.79 ± 5.14	34.09 ± 5.09	−3.27	**0.001**
	AI	35.39 ± 5.92	33.85 ± 4.215	−1.278	0.201
	AI-3MNT	36.06 ± 4.07	33.12 ± 5.7	−2.004	**0.045**
	χ^2^; p**	34.111; **0.000**	1.316; 0.518		
Active coping	BI	5.33 ± 1.16	6.12 ± 1.61	−2.34	**0.01**
	AI	6.24 ± 1.65	5.71 ± 1.33	−1.73	0.08
	AI-3MNT	6.58 ± 1.48	5.85 ± 1.37	−2.32	**0.02**
	χ^2^; p**	11.954; **0.003**	1.887; 0.389		
Acceptance	BI	6.21 ± 1.69	6.87 ± 1.46	−1.56	0.11
	AI	7.79 ± 0.41	6.94 ± 0.98	−3.97	**0.00**
	AI-3MNT	7.45 ± 0.71	6.82 ± 1.38	−1.95	0.051
	χ^2^; p**	27.136; **0.00**	0.068; 0.96		
Positive reframing	BI	5.09 ± 1.8	5.76 ± 1.56	−1.37	0.16
	AI	5.79 ± 1.43	5.85 ± 1.30	−0.07	0.93
	AI-3MNT	5.7 ± 1.89	5.76 ± 1.10	−0.28	0.77
	χ^2^; p**	2.069; 0.68	4.368; 0.263		
Planning	BI	5.82 ± 1.42	6.18 ± 1.26	−0.97	0.32
	AI	6.42 ± 1.62	5.79 ± 1.46	−1.68	0.09
	AI-3MNT	6.7 ± 1.35	6.09 ± 1.48	−1.75	0.07
	χ^2^; p**	6.686; **0.035**	0.263; 0.877		
Use of emotional support	BI	3.33 ± 1.72	4.76 ± 1.89	−3.3	**0.00**
	AI	4.36 ± 2.01	5.12 ± 1.71	−1.73	0.08
	AI-3MNT	4.55 ± 1.92	4.47 ± 1.77	−0.16	0.86
	χ^2^; p**	12.409; **0.002**	2.113; 0.348		
Use of instrumental support	BI	3.97 ± 1.96	4.44 ± 1.69	−1.23	0.21
	AI	4.88 ± 2.05	4.35 ± 1.53	−0.89	0.37
	AI-3MNT	5 ± 2.03	4.03 ± 1.08	−1.94	0.052
	χ^2^; p**	5.961; 0.051	1.152; 0.562		
Humor	BI	4.42 ± 2.33	4.85 ± 2.02	−0.958	0.338
	AI	4.33 ± 2.2	4.26 ± 1.62	−0.191	0.848
	AI-3MNT	4.58 ± 2.04	5.03 ± 1.89	−1.066	0.287
	χ^2^; p**	1.938; 0.38	3.436; 0.179		
Religion	BI	5,82 ± 2.18	5.18 ± 2.3	−1.164	0.244
	AI	5.88 ± 1.99	4.94 ± 2.10	−1.862	0.063
	AI-3MNT	5.79 ± 2.04	4.97 ± 2.63	−1.427	0.154
	χ^2^; p**	0.317; 0.853	3.303; 0.192		
World Health Organızatıon Well-Beıng Index (WHO-5)					
WHO-5	BI	53.69 ± 27.42	48.94 ± 20.79	−0.82	0.4
	AI	72.24 ± 25.42	56.4 ± 24.43	−2.7	**0.00**
	AI-3MNT	81.33 ± 16.26	51.8 ± 25.42	−4.65	**0.00**
	χ^2^; p**	33.564; **0.000**	3.323; 0.190		

p*Man Whitney U, p**Friedman’s Tests, BI: before the intervention, AI: after the intervention, AI-3MNT: 3 months after the intervention

In the beginning, the diabetes-related psychosocial self-efficacy analysis showed that the CT had higher evaluation scores than the IG for readiness to manage the psychosocial aspects of diabetes (3.79 ± 0.64 and 3.19 ± 0.63, respectively), dissatisfaction and readiness to change (3.81 ± 045 and 3.37 ± 0.45), setting and achieving diabetes goals (3.87 ± 0.42 and 3.32 ± 0.74) and psychosocial self-efficacy (3.82 ± 0.44 and 3.31 ± 0.54). Mann–Whitney U tests were conducted immediately after the intervention and 3 months after the intervention: significant improvements were seen in all scores in the IG compared to the CT. Friedman tests showed significant improvements over time in the IG scores for readiness to manage the psychosocial aspects of diabetes (χ^2^ = 52.452; *p*<0.001), dissatisfaction and readiness to change (χ^2^ = 41.785; *p*<0.001) and setting and achieving diabetes goals (χ^2^ = 46.934; *p*<0.001). All details are illustrated in Table 4.

The coping strategies data tested with the Mann–Whitney U test showed that the IG scores were significantly better than the CT scores (*p*<0.05) after 3 months. However, the Friedman test did not show significant improvement in the use of ineffective coping strategies in the IG (χ^2^ = 0.638; *p*>0.05). The IG effective coping strategies scores were significantly better than the CT scores after 3 months beyond the end of the intervention. Friedman tests showed a significant increase in the use of effective coping strategies (χ^2^ = 34.111; *p*<0.001) in the IG. When we analysed the Brief COPE sub-scores, the CT applied the following strategies more actively than the IG at the beginning (*p*<0.05): active coping, denial, emotional support and behavioural disengagement. After the intervention, behavioural disengagement was still more common in the CT; in contrast, acceptance and self-blame were significantly more common in the IG (*p*<0.05). For the IG, Friedman tests showed a significant decrease in the use of self-distraction (χ^2^ = -9.484; *p*<0.01), while they showed a significant increase in the use of active coping (χ^2^ = 11.954; *p*<0.01), emotional support (χ^2^; 12.409; *p*<0.002), behavioural disengagement (χ^2^ = 8.605; *p*<0.05), planning (χ^2^ = 6.686; *p*<0.05) and acceptance (χ^2^ = 27.136; *p*<0.001) in the IG. All details are illustrated in Table 4.

The pre-intervention WHO-5 scores in both the IG (53.69 ± 27.42) and in the CT (48.94 ± 20.79), using the cut-off point (< 50%), revealed a relatively poor emotional state in both groups (no significant difference). The comparison of group WHO-5 scores immediately and 3 months after the end of the intervention showed a significant increase in favour of the IG (*p*<0.05). Friedman tests showed significant changes in the WHO-5 scores in the IG (χ^2^ = 33.564; *p*<0.001) after the six-week and three-month follow-up, but there was no change in the control group (χ^2^ = 3.323; *p*>0.05). All details are shown in Table 4.

## Discussion

This single-blind, randomised controlled trial with a three-month follow-up indicated that the problems experienced in meaningful occupations were overcome by individuals with type 2 diabetes mellitus; therefore, it supported both diabetes care and ordinary lives. The results showed that diabetic individuals’ effective and ineffective coping scores significantly affect how individuals identify and prioritise activities of importance that they have difficulty performing. The individuals also showed that they were more ready to change their diabetes management behaviours and increase their psychosocial self-efficacy. Focusing on meaningful occupation problems and considering the demands and priorities of the person improved their participation and motivation to solve these problems effectively. Moreover, the intervention group improved their emotional well-being by the end of the process.

PST helps cope with stressful life experiences. The ineffective coping style was more effective in the univariate regression for individuals to identify and prioritise activities of importance, while the effective coping style was more effective in the multiple regression, which showed us the importance of the relationship between the factors that can affect individuals’ problematic activities. This shows the significance of person-centred, occupation-based, and holistic approaches that can be used for individuals with diabetes and that care about individuals’ perspectives and take into consideration multiple factors.

This study revealed that diabetic individuals had problems participating in meaningful activities. The findings obtained in this study showed that solving meaningful occupation problems increased the occupational performance and satisfaction of the individuals. In individuals with diabetes, a need for a holistic approach has emerged that includes self-care, as well as other priorities and factors that add meaning to the person’s life. Schultz and Schkade stated that occupational activities allowed individuals and therapists to benefit from meaningful action, and to meet their goals, the therapy programme should be directly related to individuals’ occupations in daily life [52]. Stevens stated that after serious illness and disability, occupational engagement encourages individuals’ natural motivation, which leads to a sense of self-efficacy in the patients who need to redesign and transform themselves [53]. Similarly, focusing on personal priorities in our study might have increased personal effort in participating in professional performance through increased personal motivation. Consequently, the overall results suggested that interventions for diabetic individuals through person-centred and occupation-based activities can increase their motivation to overcome problems and may help prevent the struggles of stressful life events and diabetes-related problems.

One of the most important results of this study was that the intervention group participants were able to overcome their own meaningful occupational performance problems with PST; therefore, they developed improved self-efficacy relative to the control group. Individuals who have difficulty solving problems in daily life are likely to experience similar difficulties in daily self-care regimens [28]. Corbin and Strauss reported that living with a chronic condition is associated with managing the effects of emotional problems and the chronic condition on daily life and their roles, as well as the symptoms and illness-related problems [54]. Bodenheimer et al. emphasised the importance of identifying and solving individuals’ problems for self-efficacy [55]. Since individuals’ illness-related perception of being powerless may affect their coping, Lorig and Holman indicated that their self-efficacy should be improved [56]. When investigating self-efficacy in self-management programmes, Packer suggested using strategies such as problem-solving and behavioural change [57]. In a one-year follow-up study based on this self-efficacy theory and emphasising problem-solving strategies, Lorig et al. reported an improvement in individuals’ self-efficacy and health conditions [58]. Gage and Polatajko suggested that the treatment must be associated with relevant personal performance accomplishments and that perceived self-efficacy in the individual would be higher if the individual was under control [11]. In our study, we think that the participants may have discovered the potential to overcome obstacles regarding participation in the occupations, and in this way, the increase in self-efficacy can be explained. We are of the opinion that the individual approach with occupation-based PST can support increased self-efficacy. Bandura stated that performance-based procedures were the most effective way to increase perceived self-efficacy [59]. All these studies highlight the importance of the self-efficacy of individuals with diabetes, and solving their problems in daily life can improve individuals’ self-efficacy in their struggle with diabetes. It should be kept in mind that increasing self-efficacy with a person-centred, occupation-based approach for individuals with diabetes can also potentially improve self-care behaviours.

Another important result in our study was the significance of the improvement in effective coping strategies in the intervention group. Shayeghian et al. applied acceptance and commitment therapy to the coping styles of individuals with type 2 diabetes mellitus and concluded that acceptance promoted effective coping [60]. Miles et al. applied a transactional model of stress and coping to understand diabetes self-management and emotion-focused coping, and they clarified that adaptive coping mediated the relationship between emotional and self-management behaviours, such as diet and exercise [61]. McCoy and Theekebs analysed 22 quantitative studies, and this systematic review showed that social support decreased emotional distress [62]. In our study, the intervention group preferred the use of emotional support, acceptance, planning and active coping strategies more, which are Brief COPE sub-parameters, after the intervention. The development of coping strategies in the individuals participating in our study may have been supported by several different factors of our study. The increase in the use of emotional support strategy may have shown that the person-centred approach enhances individuals’ effort to reach for supporting factors. Focusing on meaningful occupational performance problems of the individuals may have increased the motivation for active coping, and PST leads to the development of individual competence in planning necessary to achieve a solution. This result showed the importance of these strategies in overcoming diabetes-related and other problems identified by the individuals; therefore, we suggest that they should be considered in the treatment process.

Hajos stated that individuals’ psychological well-being is a basic component of their general quality of life [63]. Eakman showed that improved psychological well-being was related to participation in meaningful activities [12]. Frances showed the importance of meaningful activities through a study of artwork’s contributions to health and well-being [64]. In addition, McCoy and Theekebs showed that positive, problem-focused coping styles developed psychological and physical health [62]. The present study’s evaluation of the results according to the WHO-5 (< 50) cut-off point revealed that increased participation in meaningful activities led to an improvement in the mood of the intervention group. The abovementioned study results demonstrate that overcoming meaningful occupation and participation problems can improve individuals’ levels of psychological well-being. Our study results revealed the importance of participating in meaningful occupations in those with chronic diseases, such as diabetes. Thus, the development of adaptive skills for ordinary daily life should be added to the intervention approaches used with individuals with diabetes to improve well-being. Future research should be enhanced by person-centred, occupation-based interventions with a holistic perspective, emphasising problem solving to promote diabetes care and participation in ordinary life.

### Limitations

Our study had a follow-up period of only 3 months, and we believe that the intervention should be supported with a longer follow-up duration to provide a higher level of evidence. We also noted that time management could be affected by the gender and working status of the participants, which are factors we did not consider in our study. We recommend that future studies take gender roles and working status into account. Finally, we believe that the positive results of our study’s intervention should be supported by biometric parameters that show changes in blood glucose values, such as A1C.

## Conclusion

In summary, the key point of the current study was the examination of an supported by PST, which enabled participants to identify, sort and solve problem areas according to their own meaningful priorities. We concluded that person-centred intervention programmes that were designed to solve individuals’ meaningful occupational performance problems could support the development of self-care skills. We observed that it was important to approach individuals from a holistic point of view, to use a person-centred intervention programme and to provide the time and opportunities for them to experience the newly acquired skills. Finally, we claim that daily life and diabetes-related problems should not be treated separately; instead, individuals should be empowered by problem-solving skills and therapies that incorporate individuals’ priorities via meaningful occupation.

## Data Availability

The datasets generated during and/or analysed during the current study are available from the corresponding author on reasonable request.

## Abbreviations

PST: Problem-solving Therapy
BMI: Body Mass Index
COPM: Canadian Occupational Performance Measure
DES: Diabetes Empowerment Scale
WHO-5: World Health Organization Five Well-Being Index
IG: Intervention Group
CT: Control Group
